# Reduced Ventral Cingulum Integrity and Increased Behavioral Problems in Children with Isolated Optic Nerve Hypoplasia and Mild to Moderate or No Visual Impairment

**DOI:** 10.1371/journal.pone.0059048

**Published:** 2013-03-12

**Authors:** Emma A. Webb, Michelle A. O’Reilly, Jonathan D. Clayden, Kiran K. Seunarine, Naomi Dale, Alison Salt, Chris A. Clark, Mehul T. Dattani

**Affiliations:** 1 Developmental Endocrinology Research Group, Clinical and Molecular Genetics Unit, University College London Institute of Child Health, London, United Kingdom; 2 Neurosciences Unit, UCL Institute of Child Health and Developmental Vision Clinic, Great Ormond Street Children’s Hospital, London, United Kingdom; 3 Imaging and Biophysics Unit, University College London Institute of Child Health, London, United Kingdom; University of Maryland, College Park, United States of America

## Abstract

**Objectives:**

To assess the prevalence of behavioral problems in children with isolated optic nerve hypoplasia, mild to moderate or no visual impairment, and no developmental delay. To identify white matter abnormalities that may provide neural correlates for any behavioral abnormalities identified.

**Patients and Methods:**

Eleven children with isolated optic nerve hypoplasia (mean age 5.9 years) underwent behavioral assessment and brain diffusion tensor imaging, Twenty four controls with isolated short stature (mean age 6.4 years) underwent MRI, 11 of whom also completed behavioral assessments. Fractional anisotropy images were processed using tract-based spatial statistics. Partial correlation between ventral cingulum, corpus callosum and optic radiation fractional anisotropy, and child behavioral checklist scores (controlled for age at scan and sex) was performed.

**Results:**

Children with optic nerve hypoplasia had significantly higher scores on the child behavioral checklist (p<0.05) than controls (4 had scores in the clinically significant range). Ventral cingulum, corpus callosum and optic radiation fractional anisotropy were significantly reduced in children with optic nerve hypoplasia. Right ventral cingulum fractional anisotropy correlated with total and externalising child behavioral checklist scores (r = −0.52, p<0.02, r = −0.46, p<0.049 respectively). There were no significant correlations between left ventral cingulum, corpus callosum or optic radiation fractional anisotropy and behavioral scores.

**Conclusions:**

Our findings suggest that children with optic nerve hypoplasia and mild to moderate or no visual impairment require behavioral assessment to determine the presence of clinically significant behavioral problems. Reduced structural integrity of the ventral cingulum correlated with behavioral scores, suggesting that these white matter abnormalities may be clinically significant. The presence of reduced fractional anisotropy in the optic radiations of children with mild to moderate or no visual impairment raises questions as to the pathogenesis of these changes which will need to be addressed by future studies.

## Introduction

Children and adolescents with severe visual impairment (VI), secondary to varying etiologies, have a significantly increased prevalence of behavioral and social communication problems [1]–[3]. Optic nerve hypoplasia (ONH), a developmental abnormality of the optic nerves, is one of the leading causes of VI in the developed world, with a prevalence of 10.9 per 100,000 in England (2006) [4]–[6]. Children with severe or profound VI and isolated ONH are diagnosed with behavioral and developmental abnormalities at a similar rate to children with septo-optic dysplasia (SOD), who have other midline brain and/or pituitary hormone abnormalities in addition to ONH [7], [8]. However, the prevalence of behavioral difficulties in children with mild to moderate or no VI and ONH has not previously been recorded in the literature.

Previous neuroimaging studies in children with ONH have been limited to those using qualitative radiological assessments of conventional MR images and to two small studies (one and two individuals) using diffusion tensor imaging (DTI) [9], [10]. DTI is a non-invasive imaging technique which can provide quantitative indices of brain microstructure and enables the visualization of white matter microstructure [11], [12]. Since the basic principles of DTI were established in the mid-1990s many clinical investigations have found that abnormalities in the white matter tracts of the brain can be identified in a wide range of pathological conditions (e.g. multiple sclerosis, growth hormone deficiency and depression) [13]–[15]. They have also enabled the identification of defects in conditions not classically associated with abnormalities on conventional neuroimaging, such as autism [16]–[18]. There is a wide literature debating the reasons as to why children with heterogeneous visual disorders are at increased risk of behavioral and social communication difficulties. However, thus far, the underlying reason for this increased prevalence remains unknown [19]. It has, however, previously been suggested that reductions in exposure to visual social cues and visually guided experiences in children with VI may predispose them to developing social development abnormalities [20]. Whilst the increased prevalence of behavioral deficits found in children with ONH and severe VI may be related to their underlying visual impairment and reduced visual experience, we hypothesized that on the basis of the emerging literature regarding neuroimaging and social communication disorders such as autism [18], the underdevelopment of white matter tracts may also contribute to the behavioral abnormalities found in this cohort.

Previous studies investigating the prevalence of behavioral difficulties in children with ONH have all been in children with severe VI [1], [7]. Children with ONH and severe/profound VI frequently have co-morbidities which may potentially confound behavioral and DTI assessment including significant learning difficulties, attention deficit disorder, seizures, and cerebral palsy [8], [21], [22]. We therefore firstly aimed to assess whether children with isolated ONH with vision ranging from normal range acuity to mild to moderate VI and no developmental delay have an increased prevalence of behavioral problems compared to a control group of typically developing children without ONH. Secondly we aimed to perform detailed DTI studies in this cohort to identify whether specific white matter abnormalities, that may have not been identified on conventional MRI, are present that may provide neural correlates for any behavioral abnormalities identified.

## Methods

### Subjects

Children aged 1–11 years, diagnosed with isolated ONH by a pediatric ophthalmologist, pediatric neuroradiologist and pediatric endocrinologist, were recruited prospectively at presentation to the endocrine clinic and/or the developmental vision clinic at Great Ormond Street Hospital (GOSH). We investigated children with normal range visual acuity to mild/moderate reduction in visual acuity (mild/moderate VI) and normal cognition to remove the confounding effects of severe or profound VI and learning difficulties. A control group of typically developing children without ONH, matched for age and sex, were recruited concurrently as part of a separate study assessing the relationship between growth hormone deficiency and brain structure. The controls were all recruited from the tertiary referral hospital endocrine clinic to which they had been referred for investigation of their short stature. Short stature controls were used to control for the effect of having an underlying medical diagnosis needing investigation at a tertiary referral hospital which could impact on parental ratings reported on the Achenbach Child Behavior Checklist (CBCL). Investigations had led to no underlying medical condition being diagnosed in any of the short stature controls. These studies were approved by the NHS research ethics committee at GOSH. Written consent/assent was obtained from all parents/subjects according to the Declaration of Helsinki (BMJ 1991; 302∶1194).

### Definition of Clinical Phenotype

Isolated ONH was diagnosed when isolated ONH was present on structural brain MRI, in the presence of an otherwise normal brain and pituitary MRI scan, a normal height velocity, normal Insulin-like growth factor-1 (IGF-1) concentration for age and sex (defined as between −2 and +2 standard deviations) and otherwise normal endocrine investigations (Supplementary Information, Text S1). A diagnosis of ONH and laterality of disease were determined by one experienced pediatric neuroradiologist (WKC) blinded to the clinical data who reviewed all images. The diagnosis of ONH was confirmed by ocular fundus photography performed by a pediatric opthalmologist. Children presenting with learning difficulties (DQ or IQ 1.5 SD below the normative mean) or severe visual impairment (corrected acuity in the better eye of worse than 6/60 WHO criteria), and children with any other MRI brain abnormalities or pituitary hormone insufficiencies were excluded from entry into the study. Further details regarding the visual assessments performed to exclude learning difficulties and severe VI are outlined in the supplementary information (Text S2).

Idiopathic short stature was diagnosed in children with a height ≤2 standard deviations below the mean for age, a normal height velocity, normal brain and pituitary MRI, normal IGF-1 concentration for age and sex, a normal peak growth hormone in response to glucagon stimulation (>10 µg/L) and no reported visual impairment. One pediatric endocrinologist (MTD) reviewed the results of all endocrine investigations.

### Subject Characteristics

Eleven children (mean age 5.9 years, 81% males) with ONH and 24 controls (mean age 6.4 years, 68% males) were recruited. Of the eleven children with ONH, seven had bilateral ONH and four had unilateral ONH. Brain MRI was otherwise normal (including the hypothalamo-pituitary axis) in all subjects. Visual acuity in the better eye fell between 6/6 and 6/24 Snellen (6/6 in 4 children, 6/9 in 2 children, 6/15 in 2 children, 6/19 in 2 children and 6/24 in 1 child), indicating that the vision of the ONH children was within the functionally normal to mild/moderate VI range. IGF-1 and IGFBP-3 concentrations, thyroid function tests and glucose and cortisol profiles were normal in all children. Eleven children with ONH completed the developmental and behavioral assessment battery. Twenty-four controls were recruited, of whom 15 completed the developmental assessment (including IQ testing), 11 of the controls who underwent developmental assessment also completed the behavioral questionnaires. The time required to undertake the developmental assessment precluded some controls from consenting to undergo this component of the study. The behavioural questionnaire was added to the study protocol 6 months after recruitment of controls was started, therefore not all controls completed the behavioral questionnaire. Subject characteristics are summarised in Table 1.

**Table 1:** Number of participants, age and Sex for the whole group and for the group with behavioral and MRI data available.

Variable	Total group 1		P values	Behavioral group ^2^		P values
Group	ONH	Control		ONH	Control	
**Number**	11	24		11	11	
**Age (SD)**	5.9 (3.3)	6.4 (3)	0.56	5.9 (3.3)	6.8 (3.1)	0.58
**Male (%)**	9 (81)	17 (68)	0.78	9 (81)	10 (91)	0.91

Significance levels from statistical tests comparing the two sub-groups are also presented.

1 MRI only **^2^**MRI, developmental and behavioral data available.

### MRI Image Acquisition

MRI (standard brain and pituitary scan) and DTI sequences were acquired on an Avanto 1.5 Tesla scanner (Siemens, Erlangen, Germany). Echo-planar diffusion weighted images were acquired for an isotropic set of 20 non-collinear directions, using a weighting factor of b = 1000 s mm^−2^, along with a T_2_-weighted (b = 0) volume. This protocol was repeated three times in a single scan session, and the data merged together without averaging. 45 contiguous axial slices of thickness 2.5 mm were imaged, using a field of view of 240×240 mm and 96×96 voxel acquisition matrix, for a final image resolution of 2.5×2.5×2.5 mm. Echo time was 89 ms and repetition time was 6300 ms. In addition, a T_1_-weighted 3D FLASH structural image was acquired using 176 contiguous sagittal slices, a 256×224 mm field of view, a flip angle of 15 degrees and 1×1×1 mm image resolution. Echo time in this case was 4.9 ms, and repetition time was 11 ms.

### MRI Analysis

Diffusion-weighted images were initially processed using FSL software (http://www.fmrib.ox.ac.uk/fsl). Data were inspected for movement artifacts. Correction for eddy current induced distortions, brain extraction, and calculation of diffusion tensor FA maps was carried out using FSL tools. BET was run using its default parameters, although manual adjustments to the parameters were made if necessary to ensure a suitable brain extraction. Standard least-squares diffusion tensor estimation was used. The FA images were analyzed using tract-based spatial statistics (TBSS) [23], [24], an automated, observer-independent, voxel-by-voxel whole-brain between-group analysis technique. Initially, every FA image was aligned to every other one using the most representative study image as a target image, which is then affine-aligned into MNI152 standard space. The mean of all FA images was then created and subsequently thinned and thresholded at an FA value of 0.2 to create a white matter tract skeleton representing the center of the tracts common to all subjects. FA data projected onto these skeletons was then used in voxel-wise statistical comparisons using the Threshold-Free Cluster Enhancement option (which is fully corrected for multiple comparisons across space). All analyses were corrected for age and sex. FA was found to be significantly reduced in the optic radiations, corpus callosum and ventral cingulum on the TBSS analysis. In order to assess whether there was any association between FA for these structures and behavioral scores, values for the ventral cingulum, corpus callosum and optic radiation FA were extracted from the TBSS analysis by masking the mean skeleton with the appropriate structure label from the Johns Hopkins University white-matter tractography atlas [25].

### Developmental Assessment

Children aged over 6 years were assessed using The Wechsler Intelligence Scales for Children IV edition (WISC-IV); Full-Scale IQ, Verbal Comprehension Index, Perceptual Reasoning Index, Working Memory and Processing Speed indices (FSIQ, VCI, PRI, WMI and PSI respectively) were calculated (population mean = 100, SD = 15) [26]. Younger participants were assessed using the Wechsler Preschool and Primary Scale of Intelligence-Third Edition (WPPSI-III UK), with FSIQ, Verbal and Performance IQ scores generated [27]. Two ONH participants who were too young for the WPPSI assessment were assessed using the semi-standardized Reynell-Zinkin Scales (RZS) [28]. The RZS were administered as part of routine clinical assessment by clinical psychologists and pediatricians, who were experienced in assessing children with VI. Raw scores were converted to age equivalent levels from the normative values in the RZS manual that are appropriate for the child’s level of vision. Developmental Quotients (DQs) were derived from the mid-points of the age equivalent level divided by the chronological age of the child at the time of assessment.

### Behavior Assessment

The CBCL is a standard questionnaire assessment of children’s behavior using parent ratings [29]. The checklist includes 8 domains of behavior: Social Withdrawal, Somatic Complaints, Anxiety/Depression, Social Problems, Thought Problems, Attention Problems, Delinquent Behavior, and Aggressive Behavior. Each category is a compilation of observations about the child’s behavior with Likert scale values: 0 = no, 1 = sometimes, and 2 = very often; the sum of the values in each category reflects the severity of the behavior. The scores for internalising and externalising problems and total scores were also calculated. Internalising problems consist of syndrome scales for emotionally reactive behavior, anxious/depressed behavior, somatic complaints and withdrawn behavior. Externalising problems consist of syndrome scales for attention problems and aggressive behavior. For these scores, cut-offs for subclinical and clinical problems were set at the 84th and 90th percentiles, respectively, following the CBCL manual.

### Statistical Analysis

Age and sex of the two groups were compared using the unpaired Student’s *t* test and chi-squared test of equal proportions, respectively. Cognitive and behavioural assessment scores were compared using the unpaired Student’s *t* test. Partial correlations were used to assess the relationship between the ventral cingulum, corpus callosum and optic radiation FA and CBCL scores (controlled for age at scan and sex).

## Results

### Subject Characteristics

There were no significant differences between the age, cognitive level and sex of subjects and controls (Tables 1 and 2). All children were right-handed, had no abnormal neurological findings, were in the average range for cognition and were in mainstream schooling. There were no significant differences in age and sex between those children who underwent behavioral assessment and those who did not consent to behavioral assessment (Table 1). No significant group differences in full scale IQ, verbal or non-verbal indices were observed (Table 2). All study recruits underwent brain MRI and data quality was deemed to be adequate in all subjects (visual inspection by an experienced observer (CAC)). Significant motion artefact was not noted in any study participants.

**Table 2:** Cognitive and Child Behavior Checklist standard score means (SD) for the ONH and control participants (significant values p<0.05 in bold).

	ONH*	Control*	p value
Full Scale IQ	101.1 (22.5)	102.9 (7.1)	0.8
Verbal Comprehension/Verbal IQ	98.4 (22.6)	106.9 (7.4)	0.3
Perceptual Reasoning/Performance IQ	96.5 (19.5)	105.4 (9.9)	0.18
CBCL Anxious/Depressed	61.3 (11.6)	50.8 (2.4)	**0.014**
CBCL Withdrawn	63.5 (10.3)	52.5 (6.2)	**0.006**
CBCL Somatic Complaints	61 (8.2)	53.6 (10.7)	0.086
CBCL Social Problems	63.5 (10.7)	53.4 (10.1)	0.053
CBCL Thought Problems	65.8 (8.8)	51.5 (2.3)	**0.002**
CBCL Attention	68.3 (14.6)	53.1 (3.7)	**0.006**
CBCL Rule Breaking	58.6 (11.9)	52.7 (4.6)	0.151
CBCL Aggressive	61.8 (11.8)	51.3 (2.6)	**0.015**
CBCL Internalizing	63 (10.7)	51.7 (7.5)	**0.01**
CBCL Externalizing	60.6 (10.1)	51.3 (4.8)	**0.015**
CBCL Total Score	63.6 (11.4)	51.3 (6.4)	**0.006**

* 11 children with ONH underwent behavioral and IQ assessment;

* 15 controls underwent IQ assessment and 11 of the controls who underwent developmental assessment also completed the behavioral questionnaires.

### Behavior Assessment

Independent samples *t* tests revealed significantly more behavior problems (indicated by higher CBCL scores) in the ONH compared to the control group (Total scores p<0.006, Table 2). This pattern was found across most of the CBCL’s component subscales. Four out of the 11 children who underwent behavioral assessment (36%) in the ONH group had scores in the ‘clinical’ range (indicating more problems than were reported for 97% of the normative range), 1 of the 4 had unilateral ONH. One child, with bilateral ONH, had a score within the ‘borderline’ range, with the remainder reported as showing no behavioral problems. One child in the control group had a score in the borderline range with the remainder of the control participants having scores within the ‘normal’ range.

### Neuroimaging findings

**Figure 1:**
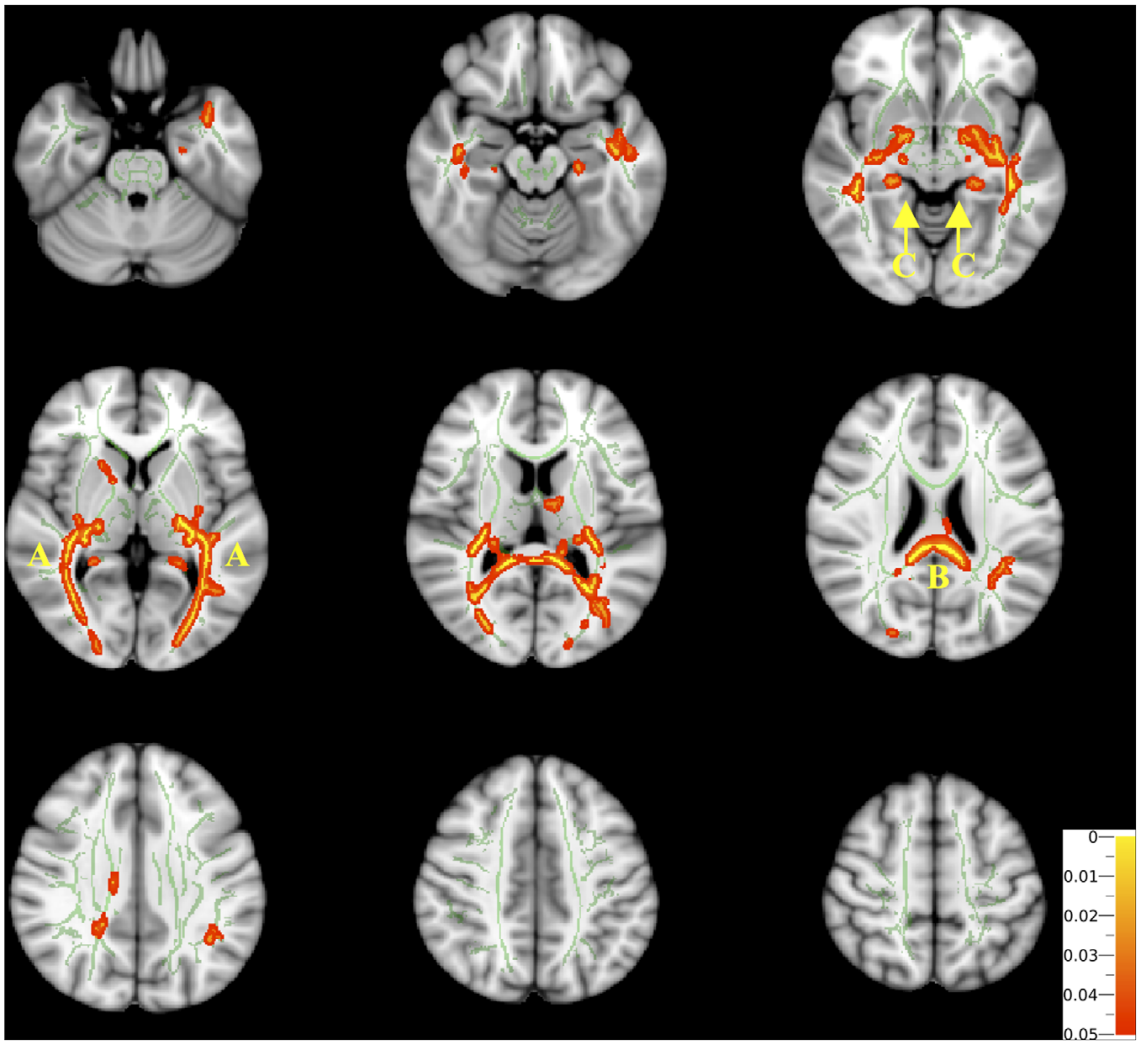
The difference in Fractional Anisotropy (FA) between children with Isolated Optic Nerve Hypoplasia (ONH) and normal controls (Tract Based Spatial Statistics Analysis comparing ONH to controls). The difference in white matter skeleton fractional anisotropy (FA) between children with isolated optic nerve hypoplasia (ONH) and controls (Tract Based Spatial Statistics Analysis comparing ONH to controls). Mean FA skeleton overlaid on the mean FA map. Regions of the mean FA skeleton in green represent areas where there were no significant differences in FA values in the ONH children compared to controls. Areas in red/yellow are regions where the FA was significantly lower in the ONH group, and can be observed bilaterally in the (a) optic radiation, (b) corpus callosum, and (c) ventral cingulum. Colour map indicates the degree of significance for red and yellow regions.

Based on the TBSS analysis FA was found to be significantly reduced in the optic radiations (bilaterally), corpus callosum and ventral cingulum (bilaterally) in children with ONH when compared to control subjects (Figure 1). There were no regions in which FA was found to be lower in the control subjects than in the children with ONH.

### Correlations between Imaging Measures and Behavioral Scores

**Figure 2:**
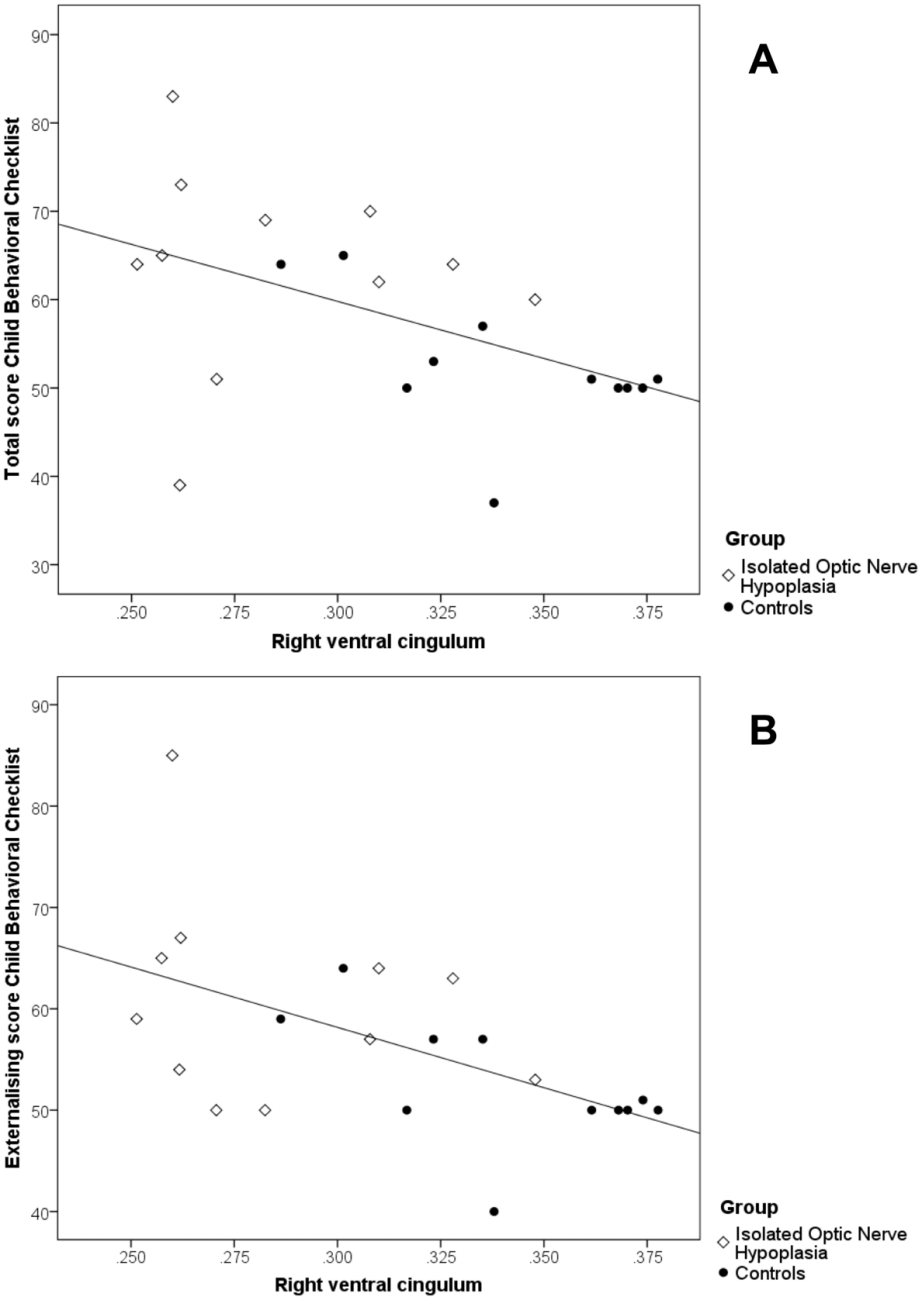
Correlations between child behavior checklist performance and ventral cingulum fractional anisotropy. Ventral cingulum fractional anisotropy (FA) was extracted from the tract based spatial statistics analysis. FA was significantly lower in the ventral cingulum in children with ONH as compared to controls. Partial correlations were used to assess the relationships between scores on the child behavioral checklist (CBCL) and right ventral cingulum FA (controlled for age at scan and sex). Higher scores on the CBCL indicate more behavioral problems. FA correlated significantly with the total (A. r = −0.52, p<0.02) and externalising score on the CBCL (B. r = −0.46, p<0.049).

Right ventral cingulum FA correlated significantly with total CBCL score (r = −0.52, p<0.02) and the externalising score on the CBCL (r = −0.46, p<0.049) (Figure 2), but not the internalising score on the CBCL r = −0.45, p = 0.056). Correlations between left ventral cingulum FA, total CBCL score and externalising and internalising scores on the CBCL did not reach statistical significance. There were no significant correlations between corpus callosum or optic radiation FA and CBCL scores.

## Discussion

This is the first study to examine behavioral difficulties and white matter integrity and their possible associations in children with isolated ONH, normal development and mild to moderate or no VI. Our findings suggest that children with ONH require behavioral assessment to exclude the presence of behavioral problems. In addition to the well-established abnormalities of the optic nerve, we have demonstrated that children with ONH also show evidence of reduced white matter integrity in the ventral cingulum bilaterally, the corpus callosum and optic radiations bilaterally. The association between white matter abnormalities in the ventral cingulum and corpus callosum have previously been identified in adults with obsessive compulsive disorder and schizophrenia [30]–[32], which suggests that the behavioral difficulties experienced by children with ONH may not solely be due to reduced visual input, but may be related to other underlying neuroanatomical abnormalities which cannot be detected on conventional brain MRI. The presence of reduced FA in the optic radiations of children with mild to moderate or no visual impairment raises questions as to the pathogenesis of these changes which will need to be addressed by future studies.

The ONH group presented with a mix of internalizing and externalizing problems, showing significant elevations in attention problems, aggressive behavior, and the anxious-depressed, withdrawn and thought problem scales of the CBCL (Table 2). Whilst we have not assessed the wider clinical significance of these behavioral difficulties, previous data demonstrate that children with a profile characterized by co-existing elevations in attention problems, aggressive behavior, and anxious-depressed scales of the CBCL have increased rates of psychiatric disorders including pediatric bipolar disorder, suicide and a high rate of adult psychopathology [33]–[35]. Our current findings highlight the importance of being alert to potential behaviour difficulties even in children with mild/moderate or no VI. Although it is not currently part of routine clinical care to perform behavioral assessment in children with ONH and mild to moderate or no VI, the incidence of clinically significant behavioral difficulties in our ONH sample (36%) is comparable to that reported previously in populations of children with severe VI (47 and 49%) [2], [3] in whom behavioral assessment is advocated.

We also identified reduced white matter integrity (FA) in the ventral cingulum and corpus callosum, likely to reflect changes in underlying brain structure [36]. FA changes can be attributed to a number of microstructural changes, including changes in axonal density, axon diameter distributions, myelin density, intra-voxel axon dispersion, unmasking due to selective tract degeneration, greater tract maturation in one tract compared to another in a crossing fibre situation in white matter, changes in cell membrane permeability, reduction in extra-cellular tortuosity as a result of increased water content in the extra-cellular space as might occur in a diffuse oedematous process and replacement gliosis. However, we expect that the axon density and myelin content explanation is the most plausible in ONH [36]. Focal reductions in FA have also been described in other paediatric conditions in which there is an increased prevalence of behavioural problems. For example, DTI studies in children with autistic spectrum disorder report reduced FA in the corpus callosum, occipitotemporal tracts, and white matter structures adjacent to the ventromedial prefrontal cortex, anterior cingulate gyrus, fusiform gyrus, superior temporal gyrus, and amygdala [37], [38], and studies in children born preterm have identified reduced FA in the corpus callosum, external capsule and the posterior aspect of the posterior limb of the internal capsule [39]. However in these other disorders the specific pattern of abnormalities we report has not been identified. The DTI findings we describe are novel in the context of ONH but perhaps unsurprising when one examines the wider literature pertaining to brain development. Murine studies have shown that axons from the cingulate cortex cross the midline prior to those of the corpus callosum, acting as pioneering axons for the corpus callosum [40], [41]. Nakata *et al* performed brain DTI in 12 individuals with agenesis of the corpus callosum to explore the hypothesis that callosal dysgenesis may represent the most obvious anatomical manifestation of a more widespread white matter developmental disorder. They identified concomitant abnormalities in the volume and structure of the ventral cingulum bundle in individuals with agenesis of the corpus callosum, concluding that this provides further evidence for a relationship between the embryonic formation of the ventral cingulum and corpus callosum [16]. Corpus callosum abnormalities are frequently found in association with ONH in SOD, an early developmental abnormality of forebrain development occurring at 4–6 weeks gestation [42]. The presence of reduced FA in the corpus callosum in the current study suggests that ONH may represent a milder end of the SOD spectrum.

Reduced ventral cingulum FA was significantly associated with CBCL scores. The cingulum provides important white matter connections within the corticolimbic neural system which is involved in regulating emotion [43]. In the current study behavioural scores were significantly associated with reduced structural integrity of the ventral cingulum. Individuals with elevated CBCL scores in childhood are at increased risk of fulfilling criteria for Diagnostic and Statistical Manual of Mental Disorders fourth revised edition [DSM-IV] diagnoses in adulthood. In adults with obsessive compulsive disorder and schizophrenia (both DSM-IV diagnoses) white matter abnormalities in the cingulum bundle and corpus callosum have been identified [30]–[32]. The finding of reduced cingulum FA in association with increased CBCL scores therefore supports our initial hypothesis that white matter abnormalities not identifiable using conventional neuroimaging methods may help to explain the increased prevalence of behavioural problems found in children with ONH.

In contrast with the two previous published studies using DTI to better understand SOD [9], [10], both of which have been performed in children classified as blind, we investigated a cohort of children with mild-moderate or no VI. Both previous studies have been in small numbers of subjects (one and two individuals respectively) and focused their analysis on the optic radiations. These studies demonstrated that children with SOD have both pre- and post-chiasmatic diffusion tensor abnormalities in the visual pathway. Previous investigations concluded that the presence of reduced FA in the optic radiations demonstrated the need for an afferent input from the retina to the lateral geniculate nucleus to stimulate normal optic radiation development [9], [10]. It is therefore noteworthy that in our cohort of children with ONH and functionally normal vision to mild/moderate VI, we have also identified significant reductions in FA in the optic radiations. This suggests that either there is some other pathophysiological process underlying the reduced structural integrity of their optic radiations, or that even small reductions in visual stimulation can affect the development of the optic radiations. A genetic etiology for SOD is currently only identified in <1% cases, and it has therefore been suggested that environmental factors including drugs, alcohol and anterior cerebral arterial supply may also be impacting on normal forebrain development in SOD (<1%) [42]. However, neither the genetic etiologies nor the environmental factors hypopthesized to impact on forebrain development would be expected to affect the development of the posterior optic radiations. We recruited a group of children with mild-moderate or no VI and therefore were unable to assess whether the neuro-anatomical and behavioral abnormalities identified related to the degree of VI present. To clarify whether the abnormal myelination/axon density in the posterior optic radiations is due to an antenatal insult or due to postnatal reductions in visual stimulation, the MRI component of this study would ideally be repeated in children at, or shortly after, birth. To further investigate the relationship between developmental visual history and early and later levels of available functional vision and the development of the posterior optic radiations this study should be repeated in children with a range of visual acuities, in the context of ONH (separating unilateral and bilateral ONH) and in VI secondary to other pathologies (e.g. retinal dystrophy).

### Conclusions

To our knowledge, this is the first study to present converging information from neuroimaging (DTI), and behavioral measures in children with ONH. Although the sample size is relatively small and has a wide age range, the sample is more homogeneous than previous studies performed in children with ONH. We have demonstrated that children with ONH, normal intelligence and mild/moderate or no VI have an increased prevalence of clinically significant behavioral problems. These are found in association with reduced structural integrity of the ventral cingulum, suggesting that they are of neuro-behavioral origin. The finding of reduced FA in the corpus callosum suggests that ONH may be part of the spectrum of SOD, a condition frequently associated with corpus callosum hypoplasia [42]. These children with mild/moderate or no VI and isolated ONH also have abnormalities in their optic radiations. This raises the question of whether abnormalities in the optic radiation in children with ONH are solely secondary to reduced visual stimulation. Further research within this population, controlling for visual levels and uni- and bilateral ONH, is required to explore the possible mechanisms affecting neural visual development.

## Supporting Information

Text S1 **Details regarding how growth rate ascertained and endocrine hormone status evaluated.**
(DOC)

Text S2 **Vision Assessment tools used.**
(DOC)
