# Supplementary material for: Reduced Ventral Cingulum Integrity and Increased Behavioral Problems in Children with Isolated Optic Nerve Hypoplasia and Mild to Moderate or No Visual Impairment
Source: PLoS One. 2013 Mar 12;8(3):e59048. doi: 10.1371/journal.pone.0059048 (PMC3595222; doi:10.1371/journal.pone.0059048)
Supplement: Text S1 — Details regarding how growth rate ascertained and endocrine hormone status evaluated. (DOC) [file pone.0059048.s001.doc]

Text S1

1. Ascertainment of growth rate and endocrine hormone status

Standing height was measured to the nearest millimetre with a stadiometer and height velocity calculated at baseline (over ≥6 months) to determine annual growth rate (serial measurements performed by one trained auxologist). Measurements of IGF-1 concentration and thyroid function were performed on all children on at least 2 occasions. All children diagnosed with ONH also underwent a 24 hr glucose and cortisol profile (two-hourly blood samples) to exclude ACTH deficiency and hypoglycaemia [1]. Mean cortisol of >145nmol/L over a 24 hour period and/or a morning peak cortisol of >175nmol/L was defined as normal [1].

1. Mehta A, Hindmarsh PC, Dattani MT (2005) An update on the biochemical diagnosis of congenital ACTH insufficiency. Clinical Endocrinology 62: 307-314.
