# Supplementary material for: Reduced Ventral Cingulum Integrity and Increased Behavioral Problems in Children with Isolated Optic Nerve Hypoplasia and Mild to Moderate or No Visual Impairment
Source: PLoS One. 2013 Mar 12;8(3):e59048. doi: 10.1371/journal.pone.0059048 (PMC3595222; doi:10.1371/journal.pone.0059048)
Supplement: Text S2 — Vision Assessment tools used. (DOC) [file pone.0059048.s002.doc]

Text S2

1. *Vision Assessment*

Visual acuity was assessed using the Sonksen LogMAR Test of Visual Acuity, Kay Picture cards (letter, symbol naming or matching), or the Keeler card (preferential looking) tests. These different acuity tests were used depending on the child’s developmental age, level of vision and ability to co-operate and communicate.
